# Assessment of Lead Exposure and Associated Risk Through Human Biomonitoring Among Urban Communities in Chennai, Southern India

**DOI:** 10.7759/cureus.73785

**Published:** 2024-11-15

**Authors:** Jayachelvi Babu, Vigneswari Aravindalochanan, Saritha Sendhil, Kalpana Balakrishnan

**Affiliations:** 1 Public Health, Sri Ramachandra Institute of Higher Education and Research, Chennai, IND

**Keywords:** blood lead levels, chennai, human biomonitoring, lead, risk assessment, southern india

## Abstract

Background: Lead exposure is a widely acknowledged risk to public health for children and adults. However, despite efforts to phase out major sources over the last several decades, estimates of the prevalence of blood lead exposures and associated risks remain poorly characterized in many low- and middle-income countries.

Objectives: This study aimed to determine blood lead levels (BLLs) and assess potential sources of lead exposure among urban communities in Chennai, Tamil Nadu.

Methodology: We obtained blood samples from a cross-sectional sample of 300 healthy individuals aged between 18 and 60. BLLs were measured using inductively coupled plasma mass spectrometry (ICP-MS) following microwave digestion. A questionnaire was administered to gather information on potential sources of lead exposure. Multivariate regression models were then developed to identify major determinants of BLLs.

Results: The mean BLL was 5.3 µg/dL with BLLs in 49% of the participants exceeding 5 µg/dL. Multiple linear regression analysis identified six predictor variables (low socio-economic status, age, male gender, proximity to industrial areas, using leaded paint and metallic cookware) that explained nearly 76% of the variance in the BLLs (F-value 82.39; p-value: < 0.001).

Conclusions: The results highlight the need for continued targeted interventions to reduce lead exposure, particularly among vulnerable populations in Chennai. Establishing greater capacities for regular biomonitoring and education on lead exposure prevention are recommended.

## Introduction

The worldwide burden of disease attributable to lead exposure is substantial and it causes 21.7 million disability-adjusted life years (DALYs) and 900,000 deaths globally [1]. Especially developing countries bear an inconsistent share of this problem, with an estimated 90% of children with elevated blood lead levels (BLLs) living in these regions [2]. The Institute for Health Metrics and Evaluation of 2019 estimated that more than 64 million Indian children had BLLs of 10 µg/dL or higher, and over 275 million Indian children had BLLs of 5 µg/dL or higher [3]. The economic costs of lead exposure are also high because it entails costs related to healthcare expenditure, loss in productivity, and diminished lifetime earnings [4]. In the last decades, intensive efforts in the world have been focused on the reduction of exposure to lead via the prohibition of leaded gasoline, the prohibition of lead-containing paints, and the control of industrial emissions. This has led to a reduction of BLLs in many countries [5]. Nevertheless, lead exposure remains the major issue worldwide, more so in developing countries like India, which have been witnessing rapid industrialization, urbanization, and lack of proper environmental legislation that have fuelled levels of lead in the environment [6].

Many Indian studies reported a high prevalence of elevated BLL in the Indian population even after phasing out of gasoline. 44.2% of school children in Delhi had BLLs over the CDC recommended limit of 10 µg/dL at the time of study [7]. A different study in Bombay and Delhi reported that 76% of children had BLLs between 5 and 20 µg/dL [8]. High BLLs were reported in many Indian studies, including children, pregnant women, and workers exposed at their workplaces [9-11]. A meta-analysis study reported that the mean BLL of the Indian population was also higher than the CDC reference levels of 5 µg/dL at 7.52 µg/dL. It further estimated that lead exposure contributes to a share of 4.6% of the total DALYs in India and thereby figured out as an almost enormous health burden load due to lead exposure within the nation.

The sources of lead exposure in India have been attributed to a combination of natural and anthropogenic emissions. Among the natural emissions are air, water, soil, and dust, while anthropogenic emissions include industrial emissions, vehicular exhaust, lead-based paints, lead-glazed ceramics, traditional medicines, and contaminated food [12]. Most of the studies have identified the sources of lead exposure to include specific lead paints in India [13] but also, soil and industrialized wastewaters [14], traditional medicines, and cosmetics such as Sindoor [15,16]. However, lead-based paints and industrial discharges remain to be the major sources of lead exposure, especially in developing countries [13]. Low exposure over a long period causes vast and permanent damage to the health of people, especially adults. Recent studies on lead have resulted in some concerns regarding the long-term health impact due to the low concentration of lead. The most vulnerable organs are the brain, kidneys, and heart, and slight alterations in blood pressure or cognitive functions may have important implications for the population at large.

Human biomonitoring is the essential method that will measure the lead internal dose, besides helping in the identification of groups at risk of adverse health effects. BLL is an established biomarker for recent lead exposure and is incorporated in most epidemiological studies around the world [11,17]. Recently, the CDC reduced its blood lead reference value for children to 3.5 μg/dL, which is lower than the previous limit of 5 μg/dL. However, the available information on lead contamination is paucity in terms of specific areas and groups, like those of Chennai. The city of Chennai is based in the Tamil Nadu state of South India with wide industrial areas of automobile, chemical, and electronics, among others, that add to the lead pollution of the environment. Studies have documented higher levels of lead in different parts of the town from air to dust samples over the years since the town has been known for lead contamination [10].

This study aims to carry out a comprehensive risk assessment of the lead exposure in the population of Chennai, India through human biomonitoring. As few data on lead exposure in Chennai are still available, and due to the growing evidence confirming the public health concern that leads present in our environment pose, this study sought to assess the risk posed by lead exposure in the population of Chennai through human biomonitoring. The specific objectives were to determine the prevalence of BLLs in the Chennai population, to compare the BLLs across different age groups, genders, and socioeconomic strata, and to identify potential sources and risk factors contributing to elevated BLLs in the study population.

## Materials and methods

Study design and participants

A cross-sectional study was conducted in Chennai, Southern India. Chennai, the capital of Tamil Nadu state, is a metropolitan located on the southeastern coast of India. It has a population density of more than seven million residents with a density of close to 26,000 people per square kilometer and comprises residential, commercial, and industrial zones The study was carried out in Chennai between 2021 and 2022. A convenient sampling method was used to enroll study participants (n=100). Participants were the outpatients who came to the medicine outpatient clinic of Sri Ramachandra Hospital (the host institution of the primary author) for trivial illnesses. Eligible participants were aged between 20 and 60 years residing in Chennai for at least five years, had never been treated for any chronic illness such as kidney, heart, and liver disorders, and had no self-reported occupational exposure to metals. Estimation of sample size for calculating a population proportion with a specified level of confidence and precision was derived from the formula. Assuming a prevalence of elevated BLLs of 20%, a precision of 5%, and a confidence level of 95%, the required sample size was calculated to be 246. The residents of Chennai were aged between 18 and 60 years and lived for at least five years prior to the study. Also, the study included written informed consent. History of Lead poisoning and having been treated for other chronic illnesses and occupational lead exposure participants were excluded from this study. Using BD Vacutainer® Tubes for Trace Element Testing (Becton Dickinson, US), venous blood samples of 3-4 mL (K2EDTA) were collected from participants. Samples were split and placed in two separate vials that were placed in -20 °C. One set was stored for analysis while the other was sent off to be stored as an added backup set. Multi-metal analysis was performed on whole blood; each batch included a duplicate sample for quality assurance purposes. The study was approved by the Institutional Ethics Committee of Sri Ramachandra Institute of Higher Education, Chennai (Ref: IEC-NI/17/JUN/60/76 dated October 16, 2017).

Data collection

Data on socio-demographics, residence history, occupation history, and information about all the potential sources of exposure to lead were obtained from a structured questionnaire. The questionnaire has four sections to collect the data on socio-demographics, residence history, occupational history, and potential sources of exposure to lead. The first three sections are obtained from the standard questionnaire used in air pollution studies [18]. The fourth section was developed based on the published literature. It was further content validated by two experts in this field. Then the questionnaire was piloted among 10 people to understand the perception of the participants regarding each question. The modified questionnaire was administered again to 20 people to test the reliability. The reliability coefficient value thus obtained was 0.91. All the questionnaires were administered in the local language by a trained enumerator. The questionnaire included socio-demographic data such as age, gender, educational details, occupation, and socio-economic status. Socioeconomic status was measured by a modified Kuppusamy scale [19], which consists of education, occupation, and monthly family income. The questionnaire also comprised the sources of the exposure including leaded paints, copper and water storage vessels made of brass, sembu, aluminum alloy, stainless steel, plastics, traditional cosmetics like Surma and kajal, and herbal medicines including residential proximity to industrial areas, levels of transportation and road traffic [14,16,20-22].

Measurement of BLLs

Venous blood (3-5 mL) was drawn from each participant using lead-free needles in the corresponding tubes through trained phlebotomists, Ethylene-diamine-tetra acetic acid (EDTA) was used as an anticoagulant. Samples were preserved at 4°C and transported to the laboratory within 24 hours after collection. It used Agilent 7800 inductively coupled plasma mass spectrometry (ICP-MS) (Santa Clara, US) with High Matrix Introduction (HMI) technology. The samples were digested [23] in a microwave using Suprapur® Nitric acid 65% from Merck KGaA (Darmstadt, Germany), with a purity of 99%, calibration, and standardization, trace element standards or CRM-NIST from Merck was applied. A reference sample was taken from Seronorm Trace Elements Whole Blood L-1 from SERO AS, Norway. To estimate the accuracy of the result, the limit of detection (LOD) of the method was defined as being at 0.2 µg/L.

Statistical analysis

The data entry was done in Microsoft Excel, and analysis was conducted with the R software version 4.3.3 (Bell Laboratories, Murray Hill, US). Descriptive statistics summarized the characteristics and distribution of BLLs among the study population. The categorical variables are displayed as frequencies and percentages, while the continuous variables are reported as mean ± SD or median and IQR. A simple linear regression was performed, and each study variable was plotted independently on BLLs. Then multiple linear regression analysis was conducted to relate the BLL in the population with the study variables that indicated a statistically significant association such as the paints applied in their residence, vessels used for cooking, proximity of residential area to industries, and other determinants such as gender, age, and socio-economic status.

## Results

A total of 300 participants were included in the study, with a mean age of 33 years (range: 18-60 years). The majority of the participants were female (66.0%) and the participants belonged to upper lower socioeconomic status 40.0% and the lower middle socioeconomic status 39%). Among the study participants, 62% had completed higher education after schooling. The blood lead reference values of ≥ 5 and 10 µg/dL are used to classify the participants with elevated BLLs. The mean, median (IQR), minimum, and maximum BLLs in each study category such as gender, age, education level, socioeconomic status, use of paints and kitchenware, conventional medicine, cosmetics, and residential proximity to industries are provided in Table 1.

**Table 1:** BLL distribution and prevalence of BLL ≥ 5 and 10 µg/dL in the study participants, Chennai, South India BLL: blood lead level

Characteristics	N	Mean (SD) (µg/dL)	Median (IQR) (µg/dL)	Min (µg/dL)	Max (µg/dL)	% ≥ 5 µg/dL	% ≥ 10 µg/dL
Overall	300	5.30 (2.30)	4.86 (3.58, 6.71)	1.11	15.30	49	2.7
Gender							
Male	101 (34%)	6.64 (2.33)	6.44 (4.55, 8.04)	2.84	15.30	72	5.9
Female	199 (66%)	4.61 (1.97)	4.23 (3.16, 5.65)	1.11	11.12	38	1.0
Age							
≤ 30	168 (56%)	5.23 (2.37)	4.77 (3.46, 6.65)	1.11	13.76	49	3.6
31 to 40	49 (16%)	5.01 (1.77)	4.37 (3.69, 6.11)	1.64	9.11	43	-
41 to 50	54 (18%)	5.73 (2.50)	5.09 (4.14, 7.11)	2.17	15.30	52	3.7
>50	29 (10%)	5.34 (2.30)	5.23 (3.22, 7.12)	2.12	9.28	55	-
Education level							
No formal education	29 (10%)	4.65 (1.61)	4.69 (3.57, 5.61)	2.12	8.24	48	-
Primary	44 (15%)	5.33 (1.89)	4.51 (4.04, 6.75)	2.57	9.20	45	-
Secondary	40 (%)	5.71 (2.64)	5.10 (4.13, 7.87)	1.64	15.30	55	2.5
Higher	187 (62%)	5.30 (2.40)	4.91 (3.47, 6.93)	1.11	13.76	49	3.7
Socioeconomic status							
Lower	31 (10%)	5.68 (2.84)	5.54 (2.26, 8.17)	2.11	11.12	55	6.5
Upper lower	121 (40%)	6.18 (2.46)	5.86 (4.52, 8.04)	1.58	15.30	71	5
Lower middle	116 (39%)	4.82 (1.75)	4.34 (3.58, 6.07)	1.11	9.61	39	-
Upper middle	29 (10%)	3.39 (0.42)	3.17 (3.11, 3.58)	3.00	4.32	-	-
Upper	3 (1%)	2.46 (0.65)	2.30 (2.11, 2.74)	1.91	3.17	-	-
Paint							
Leaded	270 (90%)	5.64 (2.16)	5.11 (4.10, 7.07)	2.12	15.30	55	3
Unleaded	30 (10%)	2.19 (0.38)	2.18 (2.11, 2.34)	1.11	2.91	-	-
Use of Vessel							
Lead-glazed metallic	226 (75.3%)	5.69 (2.22)	5.18 (4.13, 7.07)	1.11	15.30	59	2.7
Non-lead vessels	74 (24.7%)	4.09 (2.14)	3.31 (3.01, 4.28)	1.82	11.12	19	2.7
Use of traditional medicine							
Yes	24 (8%)	4.49 (0.52)	4.10 (4.08, 5.09)	3.89	5.10	42	-
No	276 (92%)	5.37 (2.38)	5.03 (3.48, 7.06)	1.11	15.30	50	2.9
Use of cosmetics							
Yes	75 (25%)	5.48 (2.39)	5.08 (3.58, 7.13)	2.12	13.21	52	4
No	225 (75%)	5.23 (2.27)	4.69 (3.58, 6.55)	1.11	15.30	49	2.2
Residential proximity to industries							
Yes	88 (29.3%)	8.11 (1.43)	8.09 (7.07, 8.52)	6.21	13.76	100	8
No	212 (70.7%)	4.13 (1.41)	4.13 (3.17, 5.09)	1.11	15.30	28	0.5

Distribution of BLLs

**Figure 1:**
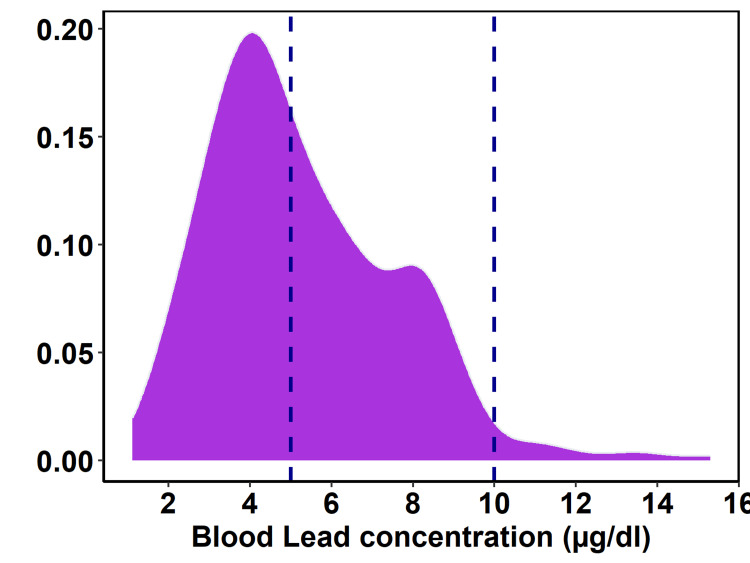
Distribution of blood lead concentration

The prevalence of BLL among 300 participants, mean 5.30 (±2.30) ranged between 1.11 and 15.30, 49% greater than 5 µg/dL and 2.7% ≥ 10 µg/dL. Within the study population, the median BLL was 4.85 µg/dL (IQR: 3.14). The distribution of BLLs among the study participants is shown in Figure 1. The greater proportions of participants (56%) were in the ≤ 30 age group, with a mean BLL of 5.23 that ranged from 1.11 to 13.76. Furthermore, 49% of the participants had ≥ 5 and 3.6% had ≥ 10 µg/dL. Within the age range of 31 to 40, 43% showed more than 5 µg/dL, although none of them exhibited ≥ 10 µg/dL. The age group between 40 and 50 years old had the most significantly increased BLLs, with a mean of 5.73 (±2.50) and a range between 2.17 and 15.30 µg/dL. Of the individuals, 52% showed greater than 5 and 3.7% showed >10 µg/dL. Notably, none of the patients over 50 years old showed >10 µg/dL. Moreover, 55% showed levels higher than 5 µg/dL.

The univariate analysis using simple linear regression showed that male gender, upper socioeconomic status, use of wall paints, lead-glazed vessel, and industrial proximity to residence were significantly associated with higher BLL. Multiple linear regression analysis results showed that 76 % of variance in the BLL can be accounted for by these six predictor variables, collectively, the F-value is 82.39; p-value: < 0.001. It also shows among the exposure variables, the residence close to the industries had a higher estimate of 3. 16 with a t-value of 19.09; p-value < 0.001. Then the usage of lead-based paints for walls showed an estimate of 2.3 with a t-value of 9.0; p-value < 0.001. The male gender showed a higher estimate compared to the female gender with a positive for BLLs (0.52 with a t-value of 3.4; p-value < 0.001) (Table 2).

**Table 2:** Blood lead level and its association with sources of exposure and socio-demographic determinants † Reference: usage of cookware (metallic lead vessels with non-lead vessels); Wall paints (Lead based paints - Chalk paints); Age - ≤30 years; Gender - Female; Socioeconomic - upper † F-value - 82.39; R2 – 0.74

Variables	Regression coefficient	95% Confidence interval		t-value	P-value
		Lower	Upper		
Gender - male	0.52	0.27	0.77	3.41	< 0.001
Age category					
31 to 40	-0.09	-0.41	0.23	-0.46	0.64
41 to 50	0.2	-0.1	0.5	1.11	0.27
>50 years	-0.03	-0.42	0.36	-0.13	0.9
Socioeconomic status					
Upper lower	-0.62	-1.81	0.58	-0.85	0.39
Lower middle	-0.06	-1.26	1.13	-0.09	0.93
Upper middle	0.55	-0.65	1.76	0.76	0.45
Upper	0.59	-0.58	1.75	0.83	0.41
Usage of cookware - lead metallic vessels	0.28	-0.2	0.77	0.96	0.34
Paints - lead based	2.3	1.87	2.72	8.96	< 0.001
Industrial proximity	3.16	2.89	3.43	19.09	< 0.001

## Discussion

This Chennai-based study among the general population showed that 49% of participants had levels above 5 µg/dL, and 2.7% of them with BLL exceeding 10 µg/dL. Males had a significantly higher prevalence of elevated BLLs compared to females. The study revealed that lead exposure is a common occurrence in the general population, the younger generation is more vulnerable to lead exposure, long long-term exposure of adults leads to irreversible chronic illnesses.

The prevalence of elevated BLLs was significantly higher among participants with upper lower socioeconomic status at 6.18 µg/dL (2.46) compared to participants with lower socioeconomic status at 5.68 µg/dL (2.84). These findings are different from those of the observations from a Korean study that observed that people from disadvantaged socio-economic backgrounds are particularly vulnerable to the adverse effects of lead exposure [24]. Similar strikingly different findings were found in the recent study conducted in Chicago, lead poisoning disproportionately affects socioeconomically disadvantaged groups, especially Black and Hispanic/Latino people [25]. Lead levels in tap water were much higher in places with lower median household incomes and lower per capita incomes, according to another Missouri study. In low-income regions, water quality deteriorates due to lead-containing corroded pipes and fixtures or the application of a protective coating to existing pipes [26]. In accordance with a Milwaukee County study, racial/ethnic makeup increased the socioeconomic disparity in mean childhood BLLs at the neighborhood level [27]. This could be attributed to the difference in the sources of lead exposure in this population and also to the variance in the time of exposure. Lead-based paints have been phased out in India. However, according to data from the NITI Aayog-CSIR study, 31% of household paints still contain lead concentrations of more than 10,000 parts per million (ppm). One of the early studies of India examined 148 paint samples from various companies that were analyzed to determine the lead content in Indian decorative paint. Lead levels in paints frequently exceed 10,000 mg/kg in India and many other Southeast Asian, African, Latin American, and European nations. For many decades to come, surfaces painted with these materials will certainly pose a major global health risk to the public [13]. One of the studies found that 39% of the paints had lead content higher than 300 ppm. Additionally, 93% of the paint samples came from the unorganized sector, while 5% came from the organized sector [28]. In this current study, more than 90.0% of the participants used paints, and also significantly correlated with BLL (p-value < 0.001).

The use of metal cookware and food and water storage containers is another source of lead exposure observed in this study is similar to that found that the lead contents in 59 pieces of cookware, including pots, woks, and pressure cookers, ranged from undetectable to 8,770 ppm (average 1,832.5 ppm) in one Bihar study [20]. El Kutry reported in his study; that certain traditional ceramics may contain lead in the glazes or embellishments on the exterior. If the pottery is not made securely, lead can leach into food and drinks prepared, stored, or served in the dishes [21]. Lead concentrations in aluminum cookware were often higher than 100 ppm. Furthermore, most of them leached more lead than was tolerated for consumption when cooking and storing in simulated environments. Lead levels in one hindalium appam pan (an Indian frying pan/wok) were high enough to surpass the pediatric limit by a factor of 14,000. Lead levels from Indian brass cookpots were also high; one cookpot's results exceeded the childhood limit by more than 1200 times. Conversely, substantially less lead is leached from cookware made of stainless steel [20]. Lead-in cookware is not currently subject to any regulatory requirements in India. More research on cookware utilizing more reliable leaching studies to determine how much lead is released during usage is required.

Lead-based paints and lead-glazed ceramics were recognized as substantial risk factors for raised BLLs in the study, consistent with earlier studies in India and other developing countries [13,29,30]. Residential proximity to industrial areas is a risk factor with which there was an association for having a BLL-elevated status, highlighting environmental lead pollution as an important population exposure source [10,11].

The study site is located in West Chennai, which extends from Poonamallee to Sriperumbudur. This region is a hub for automotive, electronics, wind turbines, engineering companies, and e-commerce. Poonamallee is more suitable for manufacture as it is centrally positioned and well-linked; it can supply the whole of Chennai city. The town has mixed sectors of residential, commercial, and industrial zones, which leads to consistently high pollution levels [30]. Participants' residences are primarily near the proximities of industrial and showed increased BLL values, also significantly correlated with a p-value of <0.001.

Strengths and limitations

The strengths of the present study include a representative sample of the population of Chennai and a higher response rate that increased the generalizability of the findings. ICP-MS has been used for the measurement of BLLs, applying matrix-matched quality control measures to validate and strengthen the exposure assessment. An extended, structured questionnaire included all the potential risk factors to identify important determinants of lead exposure among the population. A cross-sectional study design prohibits any assessment of whether the temporal relationships between the risk factors and BLLs exist. In addition, any assessment of exposure sources based only on a questionnaire is susceptible to recall bias. It also did not assess all the usual sources of lead, like occupational exposure and traditional medicine use.

Future directions

Other areas of future investigation should include longitudinal studies to better understand the temporal dynamics of lead exposure and health effects in the Indian population. Assessing other possible sources of exposure, such as occupational, traditional medicines consumption, and food ingestion, shall have a more comprehensive understanding of the exposure pathways. All interventions, such as public awareness campaigns and regulatory measures, would also be measured against the effectiveness in the future.

## Conclusions

This study provides strong evidence of the significant risk of lead exposure to the population of Chennai, India, and identifies key risk factors and vulnerable groups. It advocates for prompt concerted efforts toward lead exposure reduction and public health protection. Effective prevention of lead exposure should ideally be holistic-whether through public awareness, regulatory measures, targeted interventions, and constant biomonitoring. There is further work needed to determine the effects of various interventions, other sources of exposure, and long-term health effects of lead exposure among the Indian population.

The importance of human biomonitoring as an invaluable tool for assessing population exposure to environmental toxicants and informing public health policies is well served in this study. The integration of such data with epidemiologic and environmental data will help unravel the complicated interplay between exposure, susceptibility, and health outcomes. In summary, this study contributes to the growing body of evidence on the burden of lead exposure in India and underlines the urgent need for immediate action to reduce exposure and protect the health of the population. The findings are of paramount importance for the practice of public health and policy, and call for action by researchers, healthcare professionals, policymakers, and the public, all joining hands for a lead-free future for India and beyond.
